# α3β4 Acetylcholine Nicotinic Receptors Are Components of the Secretory Machinery Clusters in Chromaffin Cells

**DOI:** 10.3390/ijms23169101

**Published:** 2022-08-14

**Authors:** José Villanueva, Manuel Criado, Yolanda Giménez-Molina, Virginia González-Vélez, Amparo Gil, Luis Miguel Gutiérrez

**Affiliations:** 1 Instituto de Neurociencias, CSIC-Universidad Miguel Hernández, Ctra de Valencia S/N, Sant Joan d’Alacant, 03550 Alicante, Spain; 2 Departamento Ciencias Básicas, UAM Azcapotzalco, México City 00810, México; 3 Departamento de Matemática Aplicada y Ciencias de la Computación, Universidad de Cantabria, 39005 Santander, Spain

**Keywords:** chromaffin cells, exocytosis, α3β4 acetylcholine nicotinic receptor, SNAP-25, DBH, secretory machinery, particle-based methods, modeling of calcium dynamics

## Abstract

The heteromeric assembly of α3 and β4 subunits of acetylcholine nicotinic receptors (nAChRs) seems to mediate the secretory response in bovine chromaffin cells. However, there is no information about the localization of these nAChRs in relationship with the secretory active zones in this cellular model. The present work presents the first evidence that, in fact, a population of these receptors is associated through the F-actin cytoskeleton with exocytotic machinery components, as detected by SNAP-25 labeling. Furthermore, we also prove that, upon stimulation, the probability to find α3β4 nAChRs very close to exocytotic events increases with randomized distributions, thus substantiating the clear dynamic behavior of these receptors during the secretory process. Modeling on secretory dynamics and secretory component distributions supports the idea that α3β4 nAChR cluster mobility could help with improving the efficiency of the secretory response of chromaffin cells. Our study is limited by the use of conventional confocal microscopy; in this sense, a strengthening to our conclusions could come from the use of super-resolution microscopy techniques in the near future.

## 1. Introduction

Adrenomedullary chromaffin cells have been widely used as a model to study the molecular events linked to excitation−secretion coupling in neuroendocrine cells [1]. Catecholamine secretion initiates after the release of acetylcholine by splenic nerve terminals and the activation of nicotinic acetylcholine receptors (nAChRs), causing depolarization of the plasma membrane and the opening of voltage-dependent calcium channels (VDCC) [2]. The subsequent elevation of intracellular calcium is the signal leading to the transport of dense-core chromaffin vesicles to active sites, their docking, and the final release of the granule content by exocytosis [3].

Pharmacological [4] and immunological [5] approaches have shown that nAChRs in bovine chromaffin cells are of the neuronal type. A pentameric set of α3 and β4 (and probably α5) subunits appears to be the main type of nAChRs [6,7]. Binding sites for the almost irreversible antagonist α-bungarotoxin have been described in bovine chromaffin cells [8], although this toxin only inhibits a small fraction of the secretion induced by acetylcholine (Ach) [9]. This nAChR subtype contains α7 subunits, it is only expressed in adrenergic cells [10], and its possible function is still unsolved [11].

Thus, the α3β4(α5) nAChR forms a non-selective cationic channel that seems to be responsible for the depolarization that activates the secretion of catecholamines [12,13]. Moreover, the permeability of human heteromeric α3β4 nAChR to calcium has been estimated to be between 2 and 4% [14,15,16] and, in fact, the flow of calcium through the channel stimulates secretion in the absence of depolarization [17].

A key factor during secretion would be the distribution of these nAChRs in the plasma membrane, especially if they are associated or near the exocytotic machinery and, therefore, can directly enhance calcium levels at the active sites. To date, no detailed studies confirming this hypothesis in the bovine chromaffin cellular model have been reported, thus, the data presented here provide a framework to understand more precisely the role of these receptors in exocytosis and also highlight the dynamic nature of receptor distribution as a new factor contributing to the fine tuning of the secretory process in neuroendocrine cells.

## 2. Results

### 2.1. Immunocytochemical Localization of α3β4 nAChRs in the Vicinity of the Secretion Machinery

Confocal and immunocytochemical studies have shown that SNARE proteins, including the 25 kDa synaptosome-associated protein (SNAP25), can be observed to form “clusters” in the plasma membrane, and has been concluded that these “clusters” represent the secretory machinery [18]. Confocal analysis of the cultured chromaffin cells exogenously expressing an enhanced green fluorescent-SNAP-25 fusion protein (EGFP-SNAP25) demonstrated a similar clustered expression pattern that largely coincided with the immunologically detected endogenous SNAP25 clusters [19]. Based on these findings, we explored the hypothesis that endogenous α3β4 nAChRs are located in the immediate vicinity of the secretory machinery. Therefore, we used antibodies against α3 or β4 subunits to immunologically identify endogenous α3β4 nAChR structures in the cells exogenously expressing EGFP-SNAP25. We obtained polar confocal planes of each individual cell so as to obtain the measurements as close as possible to the plasma membrane area (see Section 4.2).

**Figure 1:**
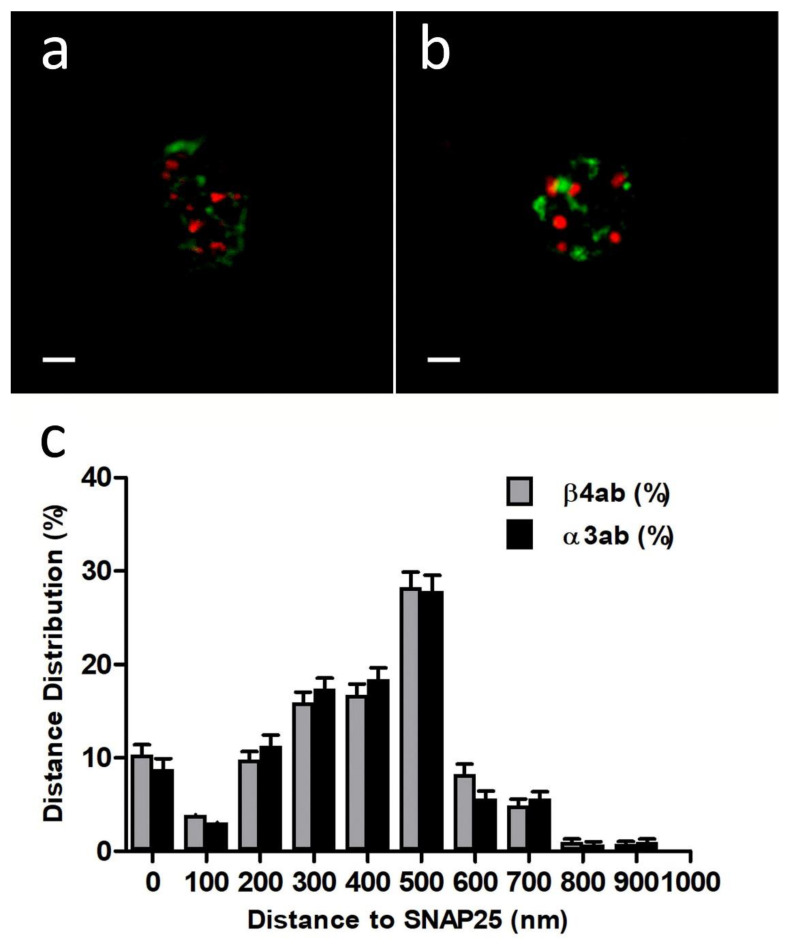
Peripheral distribution of endogenous α3 or β4 nAChR subunits to EGFP-SNAP25. (**a**) Cortical layer (TOP) of a cell expressing EFGP-SNAP25 (green) and inmunolabeled with antibody anti-β4 Atto-594 (red). (**b**) Similar image of a cell expressing EFGP-SNAP25 (green), but immunolabeled with the anti-α3 antibody and revealed with the appropriate secondary antibody Alexafluor-546 (red). (**c**) A comparison of the average distance between the centroids of each immunologically identified α3 or β4 nAChR subunits and the centroids of the nearest EGFP-SNAP25. All of the records were obtained using sequential laser excitation and acquisition. Bars indicate 1 µm.

First, we performed immunocytochemistry experiments with anti-Nicotinic Acetylcholine Receptor β4 (extracellular)-Atto-594, 48 h after transfection with EGFP-SNAP25. In all the experiments we used a sequential protocol of excitation and acquisition to avoid fluorescence crosstalk (see Section 4.3). Using free of copyright software ImageJ version 1.52i (GitHub, San Francisco, CA, USA) each cortical plane image was subjected to a threshold of 50% in both color channels before proceeding to analyze the distance between the centroid of patches comprising immunologically identified β4 subunits and the centroid corresponding to the nearest EGFP-SNAP25 clusters (Figure 1a). The distances distribution of such α3β4 nAChR identified structures in the cortical layer was analyzed (30 cells from 2 cultures), showing a population of β4-containing AChRs that was localized very close to the expressed EGFP-SNAP25 clusters with an average distance of 377.4 ± 55.9 nm (Figure 1c).

Alternatively, when similar immunocytochemistry experiments with an anti-α3 antibody (35 cells from two cultures) were performed, the distance distributions were very similar to those obtained with anti-β4 (Figure 1b,c). In fact, the resulting average distance (375.5 ± 58.5 nm) was not significantly different. These results demonstrate that both anti-α3 and anti-β4 antibodies would be equally suitable to immunologically identify the same endogenous α3β4 nAChR structures and, more importantly, that such nAChR structures would be located predominantly very close to the fusion machinery (SNAP25).

### 2.2. Distribution of Endogenous α3β4 nAChRs around Active Points upon Cell Stimulation

We employed two alternative stimulation protocols for cultured bovine chromaffin cells: either depolarization with high K^+^ or receptor activation with ACh 0.01 mM (see Section 4.5). We used either anti-β4 or anti-α3 antibodies, as they had been found to be equally suitable to identify endogenous nAChRs. To evidence exocytotic membrane patches, we used a specific sheep antibody anti-dopamine-β-hydroxylase (anti-DBH) in conditions of extracellular labeling, which were revealed with the appropriate secondary antibody AlexaFluor-488 conjugated.

For practical purposes, as not all α3β4 nAChR structures (real or simulated, see below) were close enough to the DBH exocytotic sites, we only measured the distances when they had, as a maximum, two times the average diameter calculated for DBH exocytotic structures (≤0.756 μm). In any case and for comparative purposes, the proportions of real or simulated α3β4 nAChR that were far away from this range was considered isolated. Once the random masks were generated, we analyzed the distance distributions between each DBH structure centroid and the nearest centroid of the simulated α3β4 nAChR masks.

In samples stimulated by depolarization with high K^+^, we localized 345 identified DBH exocytotic patches from 24 cells, and 22 of these points were classified as isolated. The average area of DBH and α3β4 nAChR fluorescent structures was calculated from the determined areas of the 8-bit masks of each structure obtained from the images after a threshold of 50%. The average area values for DBH exocytotic structures and α3β4 nAChRs were 0.120 ± 0.013 μm^2^ and 0.133 ± 0.014 μm^2^, respectively. These values corresponded to the diameter of a circular patch of about 0.378 ± 0.020 μm and 0.397 ± 0.021 μm, respectively. Similar data were obtained from samples stimulated by ACh 0.01mM, in which 354 identified DBH exocytotic points from 30 cells were localized, and 29 of these points were classified as isolated. The average area values for DBH exocytotic structures or identified α3β4 nAChRs structures was not significantly different from those calculated for the K^+^ stimulation assays.

In order to determine the significance of these distributions, in each analyzed sample, we simulated as many random positioned graphical masks as real identified α3β4 nAChRs we found after 50% thresholding. The randomized coordinates were obtained through a random number generator (MS-Excell RAND function) within the same graphical limits of the sample. For the simulated α3β4 nAChR masks, we used a diameter of 0.397 μm (similar to the average of the real identified α3β4 nAChR).

**Figure 2:**
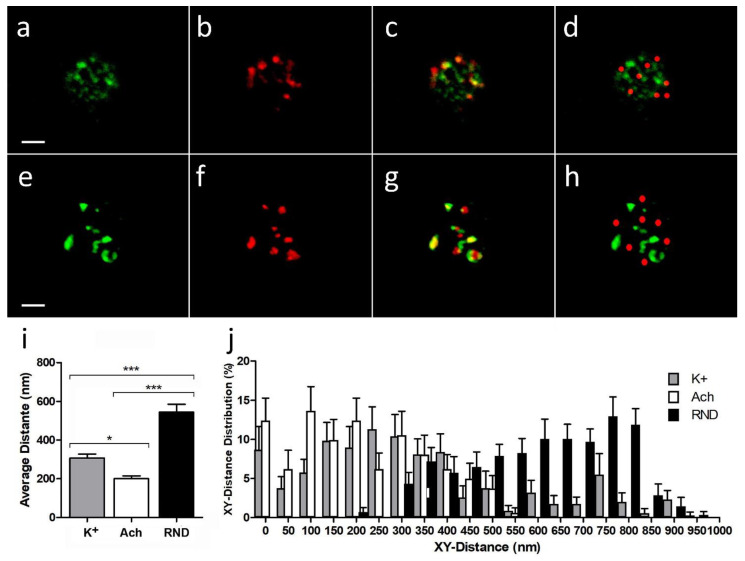
Membrane proximity between endogenous (real) or random simulated α3β4 nAChRs (red) and DBH exocytosis patches (green) upon K^+^ or ACh secretion stimulus. α3β4 nAChR real structures are apparently in contact with DBH exocytotic points more than what would be expected for a random distribution. (**a**–**d**) A chromaffin cell with both green DBH (**a**) and red α3β4 nAChR (**b**) immunolabeling in a secretion stimulus assay using depolarization with high K^+^ (see Section 4.5). Many of the real α3β4 nAChR (red) structures seem to have at least one pixel that colocalizes with DBH green patches (**c**). The random distributions of the same number of red simulated α3β4 nAChR patches are compared (**d**). (**e**–**h**) A similar experiment but using ACh as the secretion stimulus (see Section 4.5). Real (**g**) and random simulated (**h**) α3β4 nAChR structures are compared. Many of the red α3β4 nAChR structures (**f**) seem to have at least one pixel that colocalizes with DBH green patches (**g**). Significant differences are seen in the mean distance between each patch centroid and the nearest DBH centroid (**i**) (one-way ANOVA Kruskal-Wallis test (non-parametrical), K^+^ to ACh: *p* * < 0.05 moderately significative; K^+^ to RND and ACh to RND *p* *** < 0.0001 highly significative). The distribution of XY distances confirms these tendencies in each condition (**j**): under K+ stimulation (grey fill), 323 real α3β4 nAChR structures from 24 cells are measured. Under ACh stimulation (white fill), 325 real α3β4 nAChR structures from 30 cells are measured. Similar number (306) of simulated α3β4 nAChRs are measured to obtain the random distribution. Bars represent 2 μm.

When we used the high K^+^ stimulation protocol (Figure 2a–c), the average distance between DBH centroids and the nearest α3β4 nAChR centroids (307.3 ± 20.2 nm) was significantly lower than the average one when compared with a random simulation for α3β4 nAChR structures (Figure 2d) (544.6 ± 9.7 nm; *p*-value < 0.0001, Figure 2i). On the other hand, when we used the ACh stimulation protocol (Figure 2e–h), the average distance between DBH centroids and the nearest α3β4 nAChR centroids (184.0 ± 50.2 nm) was not only significantly lower than the average one when compared with a random simulation for α3β4 nAChR structures (605.7 ± 9.7 nm; *p*-value < 0.0001, Figure 2i), but also showed a moderately significant average distance reduction to that obtained for the K^+^ stimulated assays (*p*-value < 0.05). Moreover, the XY distance frequency distribution from real α3β4 nAChR structure centroids to DBH secretory locations in either K^+^ or ACh stimulation assays showed a significant shift to lower values to that for the random simulated α3β4 nAChR structures (Figure 2j).

These results suggest that, upon secretory stimuli, α3β4 nAChR structures appear significantly closer to the active secretory sites than that expected for the random distributions, and that ACh stimulation is particularly effective at moving nAChRs nearer to the secretory active sites.

### 2.3. Colocalization and Overlapping between α3β4 nAChRs and DBH Secretory Sites after Stimulation with High K^+^ and ACh

**Figure 3:**
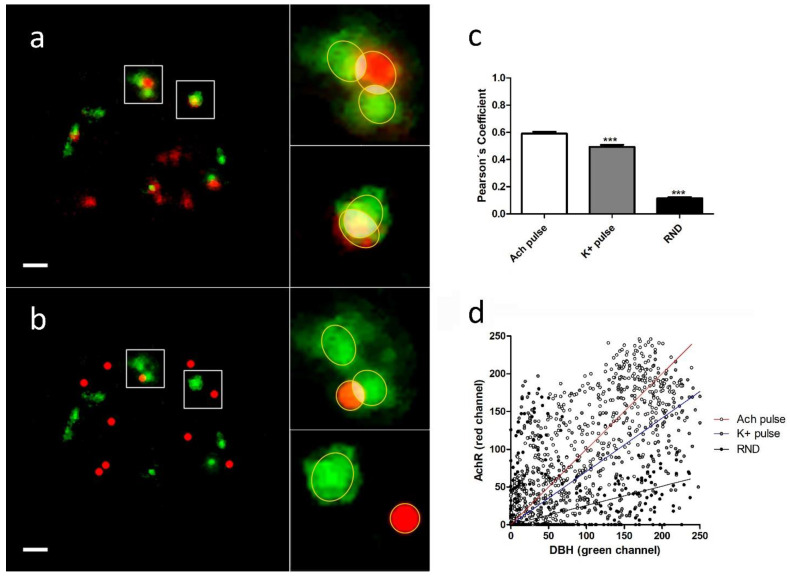
Membrane colocalization and overlapping between α3β4 nAChR (red) and DBH (green) after secretion stimulus: in each image we individually selected several regions of interest (ROIs) (**a**,**b**) and proceeded to analyze them using ImageJ JACoP complement (See Section 4.6), obtaining the values of the Pearson’s and Manders coefficients, whose averages are shown in Table 1, as well as their corresponding scatterplot graphs, whose examples are shown (**d**). The averaged Pearson’s coeficients for 50% threshold images show a significant colocalization between endogenous α3β4 nAChRs structures and the DBH sites in both stimulation conditions to those obtained for randomly simulated α3β4 nAChRs structures (Table 1 (**c**)). Furthermore, the averaged coefficients obtained for the stimulus with ACh are significantly higher than those obtained when stimulating with a high K^+^ (*p* value *** < 0.0001; Table 1). (**d**) A scatterplot or fluorogram for pixel colocalization of the red channel (AChR structures) and green channel (DBH) comparing three examples of the images obtained in each of the three analyzed conditions. Bars represent 1 µm.

The analysis of the colocalization and overlapping of the pixels between individual DBH secretion points and the nearest α3β4 nAChR structure (real or simulated) were performed using JACoP plugin (See Section 4.6). We selected each region of interest (ROI), and, after splitting both channels, the images were imported to the JACoP plugin. All of the analyzed images were subjected to 50% of thresholding and were processed to calculate Pearson’s and Manders´ coefficients for each individual selected interaction. We compared the average colocalization between exocitotic DBH patches (with the mentioned secretory stimulus) and both endogenous and randomly simulated α3β4 nAChRs (Figure 3a,b).

We found that the averaged Pearson’s and Manders’ coefficients were significantly lower when we used randomly simulated structures than when we used both stimulus conditions. Furthermore, the averaged coefficients obtained with ACh were significantly higher than those obtained with a high K^+^ (Table 1, Figure 3c).

These data indicate the existence of a certain degree of proximity between the secretory events and the endogenous α3β4 nAChRs. Furthermore, the fact that direct stimulation of the receptors with ACh implies a higher degree of colocalization between receptors and secretory sites compared with the same situation under a depolarizing stimulus (high K^+^), could suggest an active role for AChR dynamics in the regulation of the secretory process in bovine chromaffin cells.

### 2.4. Modeling of Calcium Dynamics and Secretion Involving α3β4 nAChR Distributions

In which way does the distribution of nAChR affect the secretory process of chromaffin cells? In order to answer this question, we tried to model secretory dynamics considering the influence of AChR distance on the secretory machinery. 

For modeling the nAChRs, we use the seven-state model given in [20] with the parameter values given in Table 2. The values of parameters k_+b_ and k_−b_ of the model have been modified to reproduce the inactivation of the AChR current reported in [21] for bovine chromaffin cells. Using this model, we estimate between 2250-2500 as the number of activatable nAChR channels per whole cell needed to reproduce the nAChR current amplitude. This seems to agree with other estimates in the literature [22]. In addition, in our simulations, we considered that the fractional contribution of Ca^2+^ to the current flow through nAChR channels represents 2.5% of the total current [14].

For the simulation of the calcium-induced secretory response, we used the conical domain stochastic model for chromaffin cells described in [23,24]. An immobile endogenous buffer (B_endo_) with the binding and unbinding rates given in [25] and concentration (B_endo_) = 500 µM was considered to be distributed in the intracellular domain. For the secretory vesicles, which are distributed on the first slice of the discretized simulation domain, we used the non-cooperative kinetic scheme given in [25]. The binding of calcium ions to buffers and secretory vesicles is described by first-order kinetic equations. These equations were implemented in our algorithm using a probabilistic interpretation of the kinetic reactions. In our simulation algorithm, the difference between buffer and secretory vesicles was that the binding sites of the fusion machinery were located only where the vesicles were, whereas buffer molecules were uniformly distributed all over the domain.

The experimental observations described in previous sections of this paper indicate that, after direct stimulation with ACh, there is a significant increase in colocalization between nAChRs and the active points of secretion. Therefore, in order to quantify the possible impact of such a geometrical finding on the exocytotic response of chromaffin cells, we took advantage of the geometrical flexibility of our modeling approach to evaluate the effect that the proximity of the nAChR channels to the vesicles could have on the dynamics of catecholamine release in chromaffin cells. In particular, we considered two different types of geometrical nAChR channels-vesicles configurations: (a) random configurations (vesicles and nAChRs are randomly distributed on the first slice of the simulation domain) and (b) colocalized configurations (nAChRs are first randomly distributed and then vesicles are placed in the simulation domain as close as possible to the nAChR channels).

**Figure 4:**
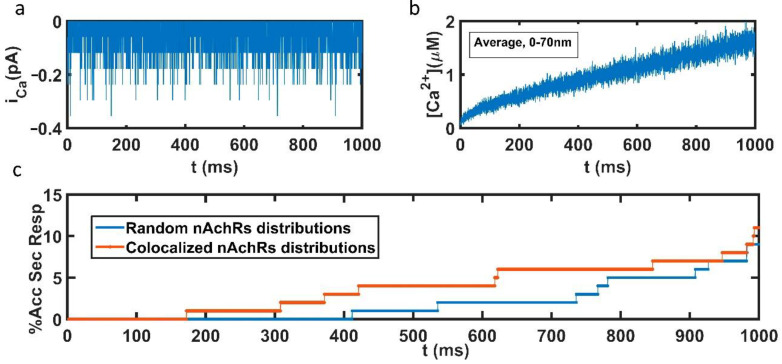
Results of the simulation of a pulse lasting 1 s. (**a**) Calcium current that enters the cell through nAChRs during the pulse (**b**). Average calcium concentration obtained at distances between 0–70 nm to the cell membrane (**c**). Time course of the accumulated secretory response (as a percentage) obtained with random and colocalized configurations of nAChR secretory vesicles.

In Figure 4, some of the results obtained with a pulse lasting 1 s are shown. We assumed nAChR = 7 nAChR channels distributed on the base of the cone (our simulation domain) and a concentration of ACh of 100 µM. Figure 4a shows the calcium current that enters the cell through nAChRs during the pulse. Figure 4b shows the average calcium concentration obtained at distances between 0–70 nm to the cell membrane: as expected, this concentration was small in comparison with the typical values of calcium concentration that enter through voltage-dependent calcium channels (VDCC) [23]. However, although small, this calcium concentration is able to trigger exocytosis, as can be seen in Figure 4c. The time course of the accumulated secretory responses (as a percentage) obtained with random and colocalized nAChRs-secretory vesicles configurations is shown. The average of five simulations corresponding to each type of geometrical configuration was considered in the calculations. As can be seen, a faster response was obtained with the colocalized configurations, which suggests that colocalization between nAChRs and the active points of secretion could help at improving the efficiency of the secretory response of chromaffin cells, especially during its initial phase. 

## 3. Discussion

Heteromeric α3β4 nAChRs are the dominant subtype of nicotinic receptors in bovine chromaffin cells. They are involved in the activation stage of the catecholamine secretion process [6,7], mainly by activating voltage-gated calcium channels (VDCC). Although nAChRs are permeable to Ca^2+^ ions and, in fact, Ca^2+^ entry through nAChRs is able to evoke exocytosis in the absence of depolarization [17], the contribution of nAChRs to the net input current of Ca^2+^ is only 2–4% [14]. Consequently, VDCC is the main source of intracellular Ca^2+^ necessary to trigger exocytosis in bovine chromaffin cells. However, further experiments in voltage-clamped bovine adrenal cells have suggested a two-step model for nAChR actions. First, light Ca^2+^ entry would keep the vesicles loaded, especially in the vicinity of the secretory machine and, accordingly, the cells would be ready to respond with a massive secretion of catecholamine upon the step of depolarization that would activate Ca^2+^ entry through VDCC [21]. Thus, in a setting in which α3β4 nAChR clusters are located in the vicinity of the secretory fusion machinery as our study suggests (Figure 1), the secretory process, especially the first phase mentioned above, would be clearly optimized.

A subsequent question is how can α3β4 nAChRs associate with other components of the secretory fusion machinery. Our previous studies have evidenced that SNAP25 or other target-soluble NSF attachment protein receptors (t-SNAREs) are associated with the edges of the cytoskeletal cages in chromaffin cells [26,27]. Moreover, these t-SNARE microdomains are colocalized with patches formed by VDCC [18], also located at the edge of the cytoskeletal cortical structures [26]. According to the data shown in the present study, α3β4 nAChR clusters were located very close to active secretory sites formed by t-SNARE proteins and, therefore, near the cortical structures of VDCC associated through F-actin, which are located below the plasma membrane in these neuroendocrine cells. Thus, this cytoskeletal structural element would be responsible for redirecting to specific membrane regions for voltage-dependent calcium channels and microdomains of the secretory machinery [28,29], as well as nAChRs.

In addition, this study has shown that upon stimulus, α3β4 nAChR clusters were located even closer to the active sites for exocytosis. This proximity proved to be much more significant than that obtained assuming a random location. Interestingly, when the physiological stimulation with ACh was performed, the degree of colocation between α3β4 nAChR groups and secretion events was significantly higher than that obtained after a potassium depolarizing stimulus (Figure 2 and Figure 3, Table 1), suggesting a dynamic role for nAChRs, not only at an initial step during the actual exocytotic process (see modeling in Figure 4), but also if successive physiological stimulations are required. In the latter case, nAChRs would already be at the vicinity of the exocytotic active sites, so that a faster and/or more efficient response would be achieved. Recently, it has been shown in human chromaffin cells that repeated ACh stimulation potentiates nAChR currents and prevents nAChR desensitization, in a process that depends on α3β4 and α7 nAChRs [30]. It seems evident that this potentiation, together with the increased proximity between α3β4 nAChR clusters and the active sites of exocytosis that we observed, could result in a further enhancement of the secretory process.

The impact of the observed location of α3β4 nAChRs was evaluated using mathematical models of secretory behavior based on Monte Carlo algorithms, which previously predicted that faster exocytotic responses would result from the association of the secretory machinery (SNARE) with calcium channels at the edges of the cortical cytoskeletal cages [27,28]. Modeling reinforced our findings, as colocalization between nAChRs and active points of secretion favored the secretion process, especially at its initial phase (Figure 4c). It is at this early phase where the slight entry of Ca^2+^ through nAChRs mentioned above [21] can favor vesicle coupling at active sites, leading to the fast release of a “ready-to-release” secretory component [31,32,33].

The connection of constituents of the secretory machinery with the cortical cytoskeleton of F-actin, including calcium channels and SNARE proteins, seems to define a special cytoarchitecture that shapes secretory kinetics. Now, α3β4 nAChR clusters moving nearer to the fusion machinery in a dynamic way, a process that was enhanced during cell stimulation, constitute a new factor to be taken into account in order to further understand how the fine tuning of the secretory response is shaped.

## 4. Methods and Materials

### 4.1. Chromaffin Native and Transfected Cells Cultures

Chromaffin cells were isolated from bovine adrenal glands by collagenase digestion and they were further separated from the debris by centrifugation on Percoll gradients. After washing, they were resuspended with Dulbecco’s Modified Eagle’s Medium (DMEM) (Sigma Madrid D-5796, stored 4 °C) to obtain a cell suspension, as described elsewhere [30].

Aliquots of the previously obtained cell suspension were used directly for both native cell culture or for transfection with the appropriate DNA construct (see below).

Native isolated cells were plated in poly-l-lysine (Sigma-Aldrich (St. Louis, MO, USA), P5899), treated coverslips in 35 mm Petri dishes (Costar, Washington, DC, USA) and maintained in DMEM supplemented with 10% fetal calf serum, 10 μM cytosine arabinoside, 10 μM 5-fluoro-2′-deoxyuridine, 50 IU/mL penicillin, and 50 μg/mL streptomycin, at a density of 150,000 cells/cm^2^. The cells were transferred to a culture incubator at 37 °C 5% CO_2_ until use.

To obtain transfected cells, we used the electroporation kit Amaxa’s system nucleofector II ^®^ system (Lonza (Basel, Switzerland), AAD-1001) with the mammalian primary neuronal cells protocol according to the manufacturer´s instructions (Lonza, VVPG-1001) (Program O-005, Amaxa GmbH, Koehl, Germany).

After electroporation, the transfected cells were plated using the same protocol described before, and were maintained in a culture incubator at 37 °C 5% CO_2_ until use. The fluorescence of the cells was studied 24 to 48 h post-plating using *SNAP25 DNA* construct.

The pEGFP-SNAP25 construct was obtained from cDNA corresponding to the SNAP25a isoform cloned into the XhoI and BamHI site of the pEGFP-C3 expression vectors (Clontech, Palo Alto, CA, USA) in order to express this protein in the frame C-terminal to EGFP [34].

### 4.2. Confocal Microscopy Studies of Fluorescent Structures

Fluorescence was visualized with an Olympus Fluoview FV300 confocal laser system mounted on an IX-71 inverted microscope incorporating a 100× UPlanSApo oil immersion objective. We used the sequential mode to register the distinct signals with a 488 nm argon ion at 40 mW to excite EGFP and a 543 nm He/Ne 10 mW for Atto-594 or AlexaFluor 546 (both lasers from Melles Griot, Carlsbad, CA, USA). The location of EGFP-SNAP25 expression was checked by confocal microscopy 24 to 48 h after transfection. Immunocytochemistry labeling of nAChR and DBH structures was monitored by using the same sequential acquisition protocol.

Typically, confocal microscopy planes were obtained from the upper external (TOP) or lower (BOTTOM) area of each individual cell in order to perform quantitative measurements in the area of the membrane [18]. Records acquired in the central plane of the cell (sagittal) were only for qualitative purposes as a visual confirmation of the apparent existence of peripheral label in the cells.

### 4.3. Immunocytochemistry of α3β4 nAChR under Basal Conditions

We revealed the immunological location of the endogenous β4 subunits using a rabbit polyclonal antibody anti-Nicotinic Acetylcholine Receptor β4 (extracellular)-Atto-594 (alomone Labs, Cat #: ANC-014-AR; Lot: ANC014ARAN0150, Jerusalem, Israel). Both native cells and those expressing EGFP-SNAP25 were washed three times with PBS 1% BSA at 4 °C, and they immediately underwent immunochemistry using the suggested manufacturing procedure for extracellular labeling, followed by a standard paraformaldehyde cell fixation protocol (11).

To confirm the localization of the endogenous α3 subunits, we employed another primary rabbit polyclonal antibody anti-Nicotinic Acetylcholine Receptor α3 (abcam, Cat #: ab151580; Lot: GR123557-8, Cambridge, UK). In this case, we proceeded to fix and permeabilize the cells before antibody incubation overnight at 4 °C in constant agitation. The next day, we performed PBS washes for 10 min and the cells were incubated with an AlexaFluor 546 goat anti-rabbit IgG secondary antibody (Invitrogen (Waltham, MA, USA), Cat # A11035, Lot: 898238) for 2 h at room temperature. The cells were conserved in PBS at 4 °C until they were visualized by confocal microscopy.

### 4.4. Immunochemistry of α3β4 nAChR under Stimulation Conditions

For the anti-β4 immunochemistry assays, we initially proceeded using the extracellular labeling protocol described in the preceding section; however, unlike previous procedures, we performed stimulation protocols before cell permeabilization and fixation. After primary antibody incubation, they were washed twice with Krebs/HEPES (K/H) basal solution containing (in mM): NaCl, 134; KCl, 4.7; KH_2_PO_4_, 1.2; MgCl_2_, 1.5; CaCl_2_, 2.5; glucose, 11; ascorbic acid, 0.56; and Na-HEPES, 15, pH 7.4 at 37 °C, and we then performed the stimulation protocol.

In the case of anti-α3 primary antibody, we proceeded firstly by performing the stimulation protocol and then, after a standard permeabilization and fixation process in Dulbecco’s Modified Eagle’s Medium (DMEM), we performed the immunocytochemical process (see below).

We designed two alternative stimulation protocols:

**Depolarization stimulating protocol:** cells were stimulated using a depolarizing solution Krebs/HEPES (High K^+^) (59 mM; obtained by isosmotically replacing NaCl by KCl) at 37 °C for 5 min. Quickly, the cells were washed with a calcium-free medium at 4 °C (to prevent endocytosis) for 3 min, and they were incubated in the same previous inmunolabelling conditions with a sheep polyclonal anti-Dopamine Beta Hydroxilase (DBH) antibody (abcam, Cat #: ab19353; Lot: GR287731-8 (N/A), Cambridge, UK) for 2 h before the standard paraformaldehyde fixation protocol [18,19]. Then, the fixed cells were revealed using an Alexafluor 488 Donkey anti-sheep IgG secondary antibody (abcam, Cat #: ab150177; Lot: GR3210700, Cambridge, UK). The cells treated previously with anti-β4-Atto-594 were washed three times with PBS and conserved at 4 °C. On the other hand, the rest of the stimulated cells were incubated with the anti-α3 primary antibody and were revealed with the AlexaFluor 546 goat anti-rabbit IgG secondary antibody (see Section 2.4). All of the cells were stored at 4 °C until use.

**Acetilcholine 0.01 mM stimulation protocol:** the cells were stimulated using Krebs/HEPES ACh stimulating solution containing (in mM): (ACh 0.01; NaCl, 134; KCl, 4.7; KH_2_PO_4_, 1.2; MgCl_2_, 1.5; CaCl_2_, 2.5; glucose, 11; ascorbic acid, 0.56 and Na-HEPES, 15, Ph 7.4.) at 37 °C for 5 min. Quickly, the cells were washed with calcium-free medium at 4 °C (to prevent endocytosis) for 3 min, and were incubated in the same previous immunolabelling conditions with the same (DBH) antibody for 2 h before the standard paraformaldehyde fixation protocol. The rest of the procedure was as described in the depolarization stimulating protocol.

### 4.5. Image Analysis

Image analysis was performed using the free of copyright software ImageJ version 1.52i (GitHub). Each individual image of two channels was analyzed to measure the distances between the centroids of the different structures identified in order to compare their relative distribution. When we wanted to analyze the colocalization and overlapping of the pixels between individual nearby structures, the ImageJ JACoP complement [35] was used, obtaining the Pearson and Manders coefficients between such structures for each region of interest (ROI) studied. In all of the cases, the data were subsequently processed with GraphPad Prism5.01 software (San Diego, CA, USA) and the graphics and images were fitted to appropriate dimensions for the final figures with Adobe Photoshop CS5 version 12.0.4 (Adobe Inc., San Jose, CA, USA).

### 4.6. Statistical Analysis

To compare populations, we first tested whether the distributions of the data adjusted well to a Gaussian distribution using the Kolmogorov−Smirnov test. If the data showed an appropriate fit, we tested whether the variances could be considered equal using a test based on Snedecor’s F distribution. If at least one population was not normally distributed, we compared the medians using a one-way ANOVA Kruskal−Wallis test (non-parametrical). For normal distributions with equal variances, we used Student’s *t* test to test the medians, and if the variances were different, we compared them using the Welch correction. Statistical tests were performed with GraphPad Prism 5.01 (San Diego, CA, USA).

### 4.7. Modeling of Calcium Buffered Diffusion

Our modeling approach is based on previous works for chromaffin cells [23,24]. We used a particle-based theoretical method (in which the fundamental variables are the number of ions and buffers) to describe the dynamics of calcium buffered diffusion in the cytosol of chromaffin cells. The basic elements in our model are as follows: (a) an adequate choice of the cellular domain and discretization, (b) a model for the entry of calcium ions through the nAChR channels, (c) the probabilistic description of 3D diffusion, and (d) the probabilistic description of the first order kinetic reactions associated with the buffering processes and exocytotic response.

As the simulation of the whole cell will take a very long time, a conical domain is a good choice to describe buffered diffusion in the submembrane domain of spherical cells (which is the case of chromaffin cells in a good approximation). The base of the cone represents the membrane of the cell in which nicotinic acetylcholine receptor channels are distributed. In our simulations, we used a conical domain with a base with a 1 μm radius and height of 5 μm, which was also the choice in [23,24]. The conical section reached the center of the prototype chromaffin cell. At the boundaries of the cone, we assumed that ions bounced back when they encountered a wall. On the lateral sides, this was equivalent to considering that the outgoing flux of the ions was equal to the incoming flux, which was a reasonable assumption because of the symmetry of the problem. An orthogonal 3D regular grid mapped the domain of the simulation with a distance between grid points of Δx = 70 nm.

## Figures and Tables

**Table 1:** Colocalization and overlapping coefficients between DBH and α3β4 nAChR (endogenous and randomly simulated) after both secretion stimulus protocols. We used one-way ANOVA (Kruskal–Wallis non-parametric test) and Dunn’s Multiple comparison test (See Section 4.7). All three coefficient comparisons were highly significant: ACh stimulus vs. K^+^ stimulus *p* value *** < 0.0001; ACh stimulus vs. RND *p* value *** < 0.0001; K^+^ stimulus vs. RND *p* value *** < 0.0001.

	Pearson’s Coefficient	Manders’ M1 Coefficient	Manders’ M2 Coefficient
ACh stimulus	0.590 ± 0.014	0.536 ± 0.011	0.571 ± 0.016
K^+^ stimulus	0.461 ± 0.097	0.399 ± 0.016	0.418 ± 0.014
RND	0.114 ± 0.009	0.079 ± 0.004	0.093 ± 0.004

**Table 2:** Parameters used in the simulation of buffered calcium diffusion in a conical domain.

Simulation Domain (Cone)		Ca^2+^, B_endo_ and ACh	
Spatial resolution	70 nm	Ca^2+^ Basal concentration	0.1 µM
Radius of the base of the domain	1 µm	B_endo_ concentration	500 µM
Height of the domain	5 µm	Diffusion coefficient of Ca^2+^	220 µm^2^ s^−1^
Number of AChR channels	7	B_endo_ forward binding rate	5.10^+8^ M^−1^ s^−1^
		B_endo_ dissociation constant	10 µM
		ACh concentration	100 µM
ACh channel model (see [20])		Secretory vesicles	
k+	227 µM^−1^ s^−1^	Number of binding sites	3
k−	38,541 s^−1^	Forward binding rate	8.10^+6^ M^−1^ s^−1^
α	2024 s^−1^	Dissociation constant	13 µM
β	50,600 s^−1^	Fusion rate	1000 s^−1^
k_+g_	49 s^−1^		
k_−g_	512 s^−1^		
k_+b_	241.5 µM^−1^ s^−1^		
k_−b_	0.3 s^−1^		

## Data Availability

All data used and/or analyzed during the current study are available from the corresponding author upon reasonable request.
